# Immune checkpoint inhibitor-driven smooth muscle cell phenotypic modulation: a potential contributor to atherosclerotic risk associated with these therapies

**DOI:** 10.3389/fcvm.2026.1789821

**Published:** 2026-07-13

**Authors:** Abhijnan Chattopadhyay, Aminat O. Dosunmu, Darshan Reddy, Sree Dharma, Krishna Panchal, Callie S. Kwartler, Dianna M. Milewicz

**Affiliations:** Division of Medical Genetics, Department of Internal Medicine, McGovern Medical School, The University of Texas Health Science Center at Houston, Houston, TX, United States

**Keywords:** atherosclerosis, immune checkpoint inhibitors, phenotypic modulation, smooth muscle cells, statin

## Abstract

Immune checkpoint inhibitors (ICIs) have revolutionized cancer therapy but are associated with increased atherosclerotic cardiovascular disease (ASCVD) risk independent of plasma cholesterol levels. While T-cell activation after ICI treatment contributes to this risk, emerging data implicate vascular smooth muscle cells (SMCs) as contributors to increased ASCVD associated with ICIs. Here, we investigated the effect of the ICI nivolumab, a monoclonal antibody targeting programmed death 1 (PD-1), on cultured human SMCs and determined that it induced activation of heat shock factor 1 (HSF1), the principal transcriptional regulator of cytosolic stress. HSF1 activation led to increased activation of HMG-CoA reductase (HMGCR), a rate-limiting enzyme in cholesterol biosynthesis, and accumulation of cholesteryl esters. Nivolumab treatment also activated endoplasmic reticulum (ER) stress, particularly PERK signaling, and atherosclerosis-associated phenotypic modulation of SMCs. Nivolumab-induced cholesterol synthesis, PERK signaling, and SMC phenotypic modulation were reversed by neutralization and knockdown of PD-1, as well as treatment with the HMGCR inhibitor pravastatin. These results reveal that nivolumab induces HSF1-HMGCR-PERK signaling and SMC phenotypic modulation and provides a rationale for statin therapy to mitigate ICI-induced ASCVD even in normocholesterolemic patients, highlighting a potential strategy to prevent ASCVD in cancer survivors receiving ICIs.

## Abstract

Immune checkpoint inhibitors (ICIs) have revolutionized cancer therapy but are associated with increased atherosclerotic cardiovascular disease (ASCVD) risk independent of plasma cholesterol levels. While T-cell activation after ICI treatment contributes to this risk, emerging data implicate vascular smooth muscle cells (SMCs) as contributors to increased ASCVD associated with ICIs. Here, we investigated the effect of the ICI nivolumab, a monoclonal antibody targeting programmed death 1 (PD-1), on cultured human SMCs and determined that it induced activation of heat shock factor 1 (HSF1), the principal transcriptional regulator of cytosolic stress. HSF1 activation led to increased activation of HMG-CoA reductase (HMGCR), a rate-limiting enzyme in cholesterol biosynthesis, and accumulation of cholesteryl esters. Nivolumab treatment also activated endoplasmic reticulum (ER) stress, particularly PERK signaling, and atherosclerosis-associated phenotypic modulation of SMCs. Nivolumab-induced cholesterol synthesis, PERK signaling, and SMC phenotypic modulation were reversed by neutralization and knockdown of PD-1, as well as treatment with the HMGCR inhibitor pravastatin. These results reveal that nivolumab induces HSF1-HMGCR-PERK signaling and SMC phenotypic modulation and provides a rationale for statin therapy to mitigate ICI-induced ASCVD even in normocholesterolemic patients, highlighting a potential strategy to prevent ASCVD in cancer survivors receiving ICIs.

## Introduction

Immune checkpoint inhibitors (**ICI**s) have transformed cancer treatment and are the standard of care for many malignancies (1, 2). ICIs increase the risk for atherosclerotic cardiovascular diseases (ASCVD), and data indicate activation of T cells when proteins such as PD-1 (programmed death 1) are blocked contributes to this risk (3). At the same time, hypercholesterolemic PD-L1 (PD-1 ligand) knockout mice have increased smooth muscle cell (**SMC**)-positive areas in their atherosclerotic plaques, suggesting that these cells may also contribute to plaque burden with ICI treatment (4, 5).

Lineage tracing of atherosclerotic plaque cells from hypercholesterolemic mice has delineated SMC phenotypic modulation that occurs during plaque formation, which includes de-differentiation and increased expression of markers for macrophages (galectin 3, *Lgals3*), fibroblasts (fibronectin 1, *Fn1*), chondrocytes (secreted phosphoprotein 1, *Spp1*), and stem cells (lymphocyte antigen 6 complex, locus A, *Ly6a*) (6). Components of this SMC phenotypic modulation can be driven *in vitro* by exposing SMCs to exogenous cholesterol. Increasing cellular cholesterol activates endoplasmic reticulum (**ER**) stress, and specifically protein kinase R-like endoplasmic reticulum kinase (**PERK**) signaling, which subsequently recruits Krüppel-like factor 4 (**KLF4**) to drive SMC modulation to macrophage/chondrocyte-like cells (7). The impact of PERK signaling in SMCs in plaque formation *in vivo* is evident by the 80% decrease in plaque burden in male hypercholesterolemic mice with SMC-specific *Perk* deletion (8). Cholesterol-driven modulation of SMCs is also responsible for the early-onset ASCVD associated with pathogenic missense variants in *ACTA2,* the SMC-specific isoform of a-actin, which cause misfolding of mutant SMC α-actin and induction of cytosolic stress. This stress activates heat shock factor 1 (**HSF1**), which increases cholesterol biosynthesis, including augmenting the levels and activity of 3-hydroxy-3-methylglutaryl-CoA reductase (HMG-CoA reductase, **HMGCR**) and increasing cholesterol ester (**CE**) levels, thus triggering the same PERK pathway to modulate SMCs (9). In fact, simply heat shocking SMCs at 42 °C for 45 min activates HSF1, cholesterol biosynthesis and CE accumulation, and atherosclerosis-associated SMC phenotypic modulation (9, 10). The same signaling pathway underlies ASCVD associated with biallelic loss-of-function variants in the centrosomal scaffolding protein pericentrin (11). Here, we investigated whether nivolumab treatment activates the same stress pathways in SMCs, thus contributing to SMC phenotypic modulation and accelerated atherosclerosis in patients undergoing ICI therapy.

## Materials and methods

### Data availability

The raw data supporting the conclusions of this article will be made available by the authors, without undue reservation.

### Cellular treatment, immunoblotting, and quantitative real time PCR

Immortalized aortic SMCs from healthy human donors (collected in accordance to protocols approved by the Committee for the Protection of Human Subjects of the University of Texas Health Science Center at Houston) were exposed to indicated amounts of the anti-human PD-1 monoclonal antibody nivolumab (Selleck Chemicals) or human IgG4 isotype (Selleck Chemicals), with or without pravastatin as indicated, for indicated amounts of time in treatment medium – Dulbecco’s minimum essential medium (DMEM) with high glucose, containing 1% antibiotics, 10% fetal bovine serum (FBS) and 0.2% bovine serum albumin (BSA), at 37 °C in a 5% CO_2_ incubator.

#### Competition assay

WT human SMCs were seeded in 6- or 12-well plates and allowed to attach overnight. The plates were then chilled for 30 min at 4 °C to avoid internalization and designated wells were washed with phosphate-buffered saline (PBS) and pre-blocked with 10*μ*g/mL mouse IgG1*κ* isotype (Biolegend, #401401) or 10 *μ*g/mL mouse anti-human CD279 (PD-1) antibody (Biolegend, #329902) respectively in treatment medium, at 4 °C for 30 min (to avoid internalization). SMCs in parallel wells that were not pre-blocked were then washed with PBS and treated with either treatment medium only or with 10μg/mL of nivolumab or human IgG4 isotype, while the pre-blocked cells were directly treated with 10μg/mL of nivolumab, without washing off the blocking antibodies. All cells were incubated at 37 °C for 72 h in presence 5% CO_2_.

#### RNA interference

To downregulate *PDCD1* (the gene coding PD-1), human SMCs were transfected with an ON-TARGETplus Human PDCD1 siRNA SMARTPool (Horizon Discovery, L-004435-00-0005; designated si-*PDCD1*(1)), an individual siRNA (Horizon Discovery, J-004435-05-0002; designated si-*PDCD1*(2)), or a non-targeting control pool (Horizon Discovery, D-001810-10-05; designated si-Control), using Liopfectamine RNAiMAX and incubated for 48 h, following which *PDCD1* expression was assessed using qPCR.

#### Immunoblotting and antibodies

Immunoblotting was performed as described by us previously (9). The following antibodies were used for immunoblotting and functional assays Table 1:

**Table 1:** Details of antibodies used in this study.

Antigen	Vendor	Catalog #	Dilution/amount
phospho-HSF1 (Ser326) (Rabbit)	Bioss Antibodies	bs-3741R	1:500
Total HSF1 (Rabbit)	Cell Signaling Technology	4356S	1:500
phospho-eIF2*α* (Rabbit)	Cell Signaling Technology	5342S/D7D3	1:1000
Total eIF2α (Rabbit)	Cell Signaling Technology	3398S S/D9G8	1:10000
ATF4 (Rabbit)	Abcam	ab216839	1:1000
KLF4 (Goat)	RnD Systems	AF3158	1:500
GAPDH (Rabbit)	Cell Signaling Technology	2118/14C10	1:10000
Anti-human CD279/PD-1 (mouse)	Biolegend	329902	10μg/mL
Peroxidase-AffiniPure Goat Anti-Rabbit IgG, F(ab')2 Fragment Specific antibody	Jackson ImmunoResearch Labs	111-035-006	1:4000
Mouse anti-goat IgG-HRP	Santa Cruz Biotechnology	sc-2354	1:4000

Total RNA isolation and qRT-PCR were performed as described earlier, with *GAPDH* as internal control (Applied Biosciences) when using Taqman chemistry (Quantabio) and with *18S rRNA* as internal control (Millipore Sigma) when using SYBR green chemistry (Quantabio) (8, 9). SYBR green primers are provided below Table 2:

**Table 2:** List of human SYBR Green primers used in this study.

Gene	Forward primer (5’ -> 3’)	Reverse primer (5’ -> 3’)
*18S rRNA*	GTAACCCGTTGAACCCCATT	CCATCCAATCGGTAGTAGCG
*ATF4*	AAACCTCATGGGTTCTCCAG	GGCATGGTTTCCAGGTCATC
*FN1*	TGGTGGCCACTAAATACGAA	GGAGGGCTAACATTCTCCAG
*HSF1*	GGAAAGTGGTCCACATCGAG	TTCACTCTCCCGCAGGATGG
*LGALS3*	ATGGCAGACAATTTTTCGCTCC	GCCTGTCCAGGATAAGCCC
*PDCD1*	AAGGCGCAGATCAAAGAGAGCC	CAACCACCAGGGTTTGGAACTG
*SPP1*	CGAGGTGATAGTGTGGTTTATGG	GCACCATTCAACTCCTCGCTTTC

### Transwell migration assay

SMC migration in response to nivolumab was assessed using Transwell assay as described by us previously (9, 11). Six randomly chosen fields were imaged per condition using a ZOE Fluorescent Cell Imager (BioRad), and DAPI-stained nuclei were counted using ImageJ software in a blinded fashion.

### Luciferase activation assay

SMCs were transfected with HSF1 or KLF4 Cignal Reporter plasmid (Qiagen) and treated with indicated amounts of nivolumab or IgG isotype for 72 h. Luciferase activity was analyzed using a Dual Luciferase Assay kit (Promega) as described by us earlier (9, 11).

### HMGCR enzymatic activity assay

Enzymatic activity of HMGCR was estimated using a colorimetry-based HMG-CoAR activity assay kit (Abcam, ab204701) as described by us earlier (9, 11).

### Estimation of free and esterified cholesterol

Total and free cholesterol in SMCs treated with nivolumab or human IgG4 were estimated using a Cholesterol/Cholesteryl Ester Quantitation Assay kit (Colorimetric/Fluorometric, Abcam, ab65359), as described by us previously (9). Esterified cholesterol was calculated as the difference between total and free cholesterol. All concentrations were normalized to DNA concentrations.

### Proliferation assay

Cellular proliferation was assessed using a Click-iT™ Plus EdU Alexa Fluor™ 647 Flow Cytometry Assay Kit (Thermo Fisher Scientific), according to manufacturer’s instructions, as described by us previously (9).

### Assessment of apoptosis and necrosis

Apoptosis and necrosis in human SMCs following nivolumab and IgG4 exposure were assessed by flow cytometry, using a GFP-CERTIFIED® Apoptosis/Necrosis Detection Kit (Enzo Life Sciences), according to manufacturer’s instructions, as described by us previously (7).

### Statistical analyses

Cellular experiments were performed using SMCs isolated from two different donors and each individual experiment was performed three times. Data were checked for normality using GraphPad Prism 10.6.0. Normally distributed data were analyzed using Student's t-test followed by Welch's correction when comparing two groups and by two-way ANOVA followed by Tukey's multiple comparisons test when comparing multiple groups. When the data did not pass normality, they were analyzed with Mann–Whitney U-test when comparing two groups and Kruskal–Wallis test followed by Dunn's multiple comparisons test when comparing multiple groups. A corrected *p*-value of <0.05 was considered statistically significant.

## Results

**Figure 1:**
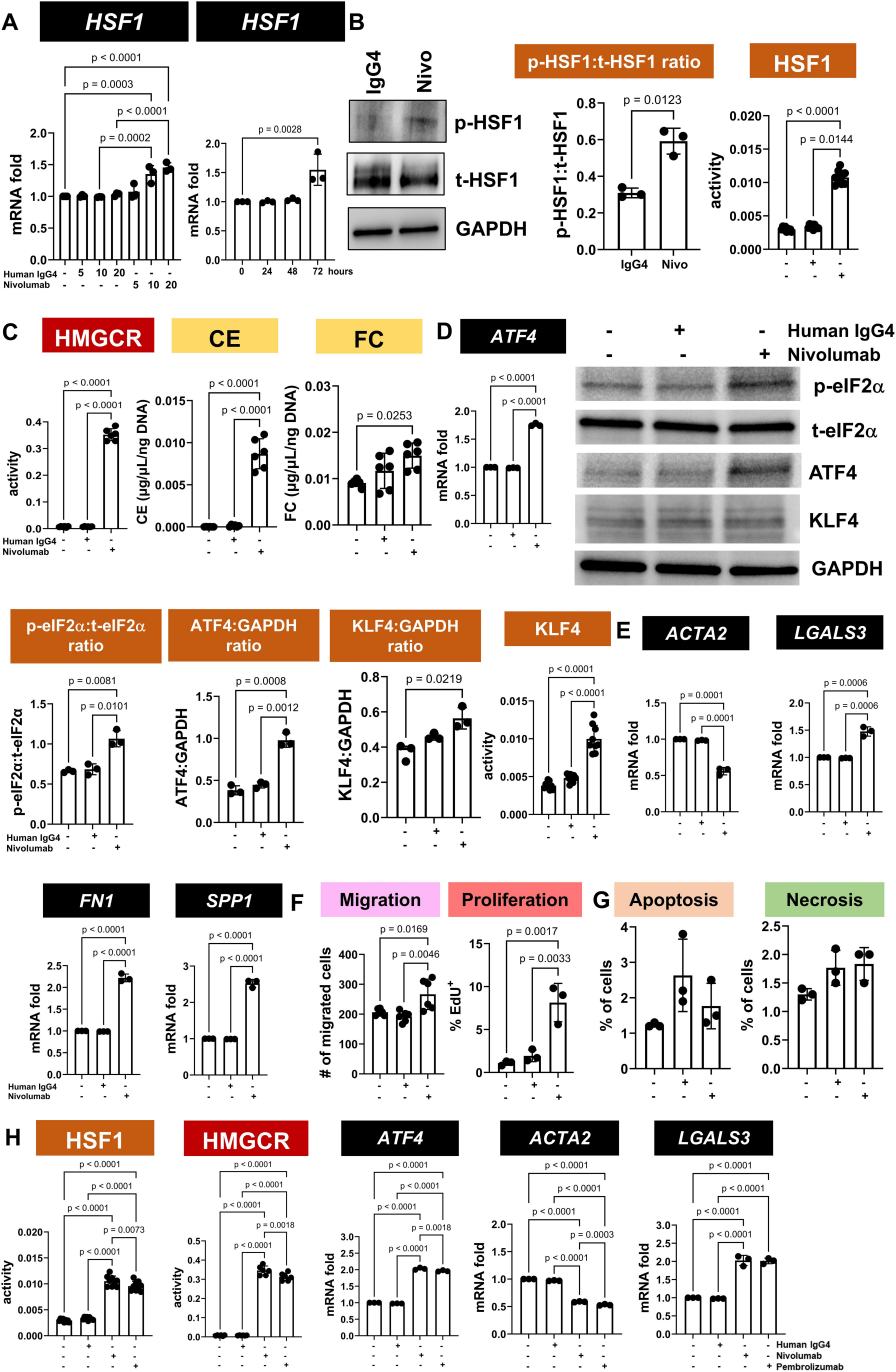
Anti-PD-1 antibodies induce cytosolic stress, cholesterol biosynthesis, PERK signaling and atherosclerosis-associated phenotypic modulation in SMCs. **(A)** Exposure to 10 *μ*g/mL or higher nivolumab for 72 h induces *HSF1* mRNA in human aortic SMCs. **(B–D)** Human SMCs exposed to nivolumab, but not human IgG4, increase SMC migration, HSF1 phosphorylation (Ser 326) and transcriptional activation, leading to enhanced HMGCR activity and increased intracellular CE levels, with a modest increase in free cholesterol levels. **(E,F)** Nivolumab exposure induces PERK signaling - as observed from increased phosphorylation of eiF2α, increased *ATF4* expression and protein levels, as well as increased transcriptional activation of KLF4 – (KLF4 protein level was not significantly increased with nivolumab treatment compared to IgG4), and phenotypic modulation, as evident from the decreased expression of the contractile marker *ACTA2*, and increased expression of the SMC phenotypic modulation markers, *LGALS3, FN1* and *SPP1.* **(G)** Nivolumab exposure did not induce apoptosis or necrosis in human SMCs. **(H)** Exposure to a second α-PD-1 therapeutic antibody, pembrolizumab, also induces HSF1 and HMGCR activation, *ATF4* and *LGALS3* expression, while downregulating *ACTA2* in human SMCs. Phosphorylated HSF1 and eIF2α were normalized to total HSF1 and eIF2α, respectively. All data passed normality except HSF1 activity **(C)** and blot quantification for KLF4 **(F)** and were analyzed by unpaired student's t-test (blot quantification in C) or 2-way ANOVA followed by Tukey's multiple comparisons test. HSF1 activity and KLF4 blot quantification did not pass normality and were analyzed using Kruskal–Wallis test, followed by Dunn's multiple comparisons test. Nivo - nivolumab, CE – cholesteryl esters, FC – free cholesterol.

Since SMCs are not canonical PD-1-expressing (encoded by *PDCD1*) cells, we performed qRT-PCR to determine its expression in immortalized aortic SMCs from healthy human donors (12). *PDCD1* amplified at cycle 25.77 ± 1.18, indicating that the gene is expressed by SMCs. To determine if ICI exposure activates HSF1 to drive SMC phenotypic modulation, immortalized aortic SMCs from healthy human donors were exposed to increasing concentrations (0–20μg/mL) of the anti-human PD-1 monoclonal antibody nivolumab or human IgG4 isotype for 72 h (note that the concentrations of nivolumab used here are lower than 66.7*μ*g/mL - the average steady-state serum levels in patients receiving 240 mg nivolumab biweekly) (13). 10 *μ*g/mL nivolumab for 72 h was sufficient to induce *HSF1* mRNA and that combination of concentration and treatment time was used for the remaining experiments in the study (Figure 1A). Nivolumab exposure leads to the following: phosphorylation of HSF1 (Ser 326) and transcriptional activation of HSF1 (Figure 1B); enhanced enzymatic activity of HMGCR and increased formation of CEs (free cholesterol levels went up modestly with nivolumab compared to baseline, but there was no statistically significant difference between cells exposed to nivolumab and IgG, Figure 1C); increased PERK signaling (increased eukaryotic initiation factor 2α - eIF2α - phosphorylation, elevated *ATF4* – activating transcription factor 4 - expression and protein levels; increased KLF4 transcriptional activation with modest increase in KLF4 protein level; Figure 1D) and SMC phenotypic modulation, including decreased expression of the contractile gene *ACTA2* and increased markers of other cell types (*LGALS3, FN1* and *SPP1*), increased SMC migration and proliferation (Figures 1E,F). Neither nivolumab nor IgG4 exposure affected cell viability, as evidenced by the lack of induction of apoptosis or necrosis (Figure 1G). Finally, to confirm whether the observed effect of nivolumab is generalizable to other ICIs, we compared it with another FDA-approved IgG4 a-PD-1 antibody, pembrolizumab, that is used in the treatment of multiple cancers (14, 15). Pembrolizumab exposure, like nivolumab, induced HSF1 activation, increased HMGCR activity and induced *ATF4* and *LGALS3* while leading to the downregulation of *ACTA2*, indicating that multiple therapeutic antibodies targeting PD-1 activate HSF1-HMGCR-PERK signaling (Figure 1H).

**Figure 2:**
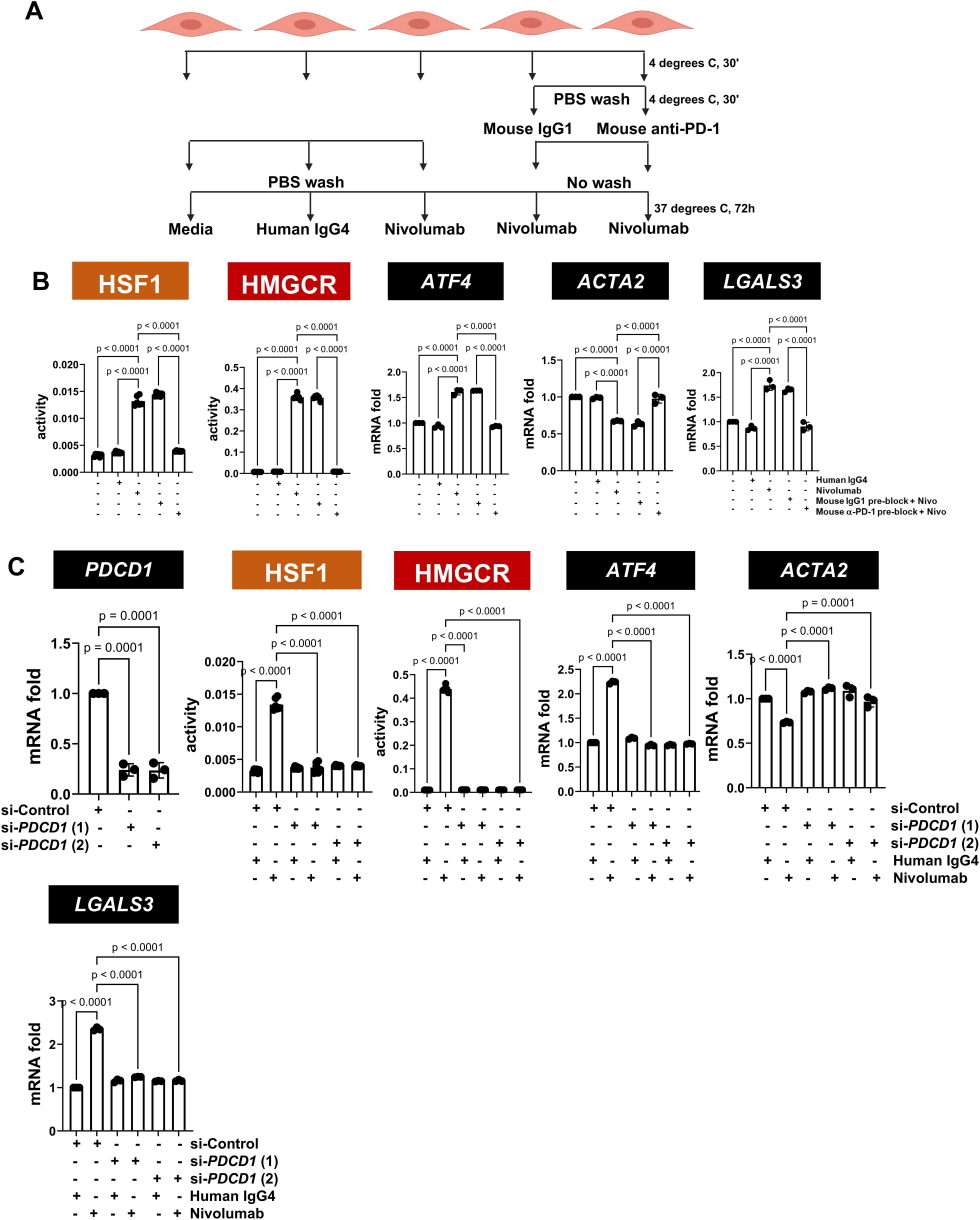
Both pre-blocking with a-PD-1 antibody and downregulation of PD-1 attenuate the effect of nivolumab on SMCs. **(A)** Experimental schematic describing pre-blocking of SMCs with IgG or α-PD-1 antibody and subsequent treatments in a competition assay. **(B)** Pre-blocking with an α-PD-1 antibody, but not the corresponding IgG isotype control, blocks nivolumab-induced activation of HSF1 and HMGCR, and the upregulation of *ATF4* and *LGALS3*, while preserving the expression of *ACTA2*. **(C)** siRNA-mediated knockdown of *PDCD1* attenuates the ability of nivolumab to increase HSF1 and HMGCR activity, induce *ATF4* and *LGALS3*, and downregulate *ACTA2*. All data passed normality and were analyzed by 2-way ANOVA followed by Tukey's multiple comparisons test. Nivo - nivolumab.

To test whether nivolumab-induced pathway activation involves PD-1 engagement, human SMCs were pre-incubated with either control IgG (mouse IgG1*κ* isotype) or a PD-1 blocking antibody (mouse anti-human) prior to nivolumab exposure. Pre-treatment with only the anti-PD-1 antibody but not the IgG attenuated HSF1 activation and induction of *HMGCR*, *ATF4* and *LGALS3*, indicating nivolumab-induced activation of downstream HSF1–HMGCR–PERK signaling and phenotypic modulation is dependent, at least in part, on PD-1 engagement in human SMCs (Figures 2A,B). To further confirm engagement of PD-1, *PDCD1* expression was downregulated using a pool of siRNAs or an individual siRNA against *PDCD1* for 48 h, following which the cells were treated with 10 *μ*g/mL of human IgG4 or nivolumab for 72 h. *PDCD1* downregulation attenuated nivolumab-induced increases in the transcriptional activation of HSF1, enzymatic activity of HMGCR, and the expressions of *ATF4* and *LGALS3*, while reversing the downregulation of *ACTA2* (Figure 2C). Taken together with the competition assay results, these results reinforce that nivolumab-induced activation of HSF1-HMGCR-PERK signaling and phenotypic modulation engages PD-1.

**Figure 3:**
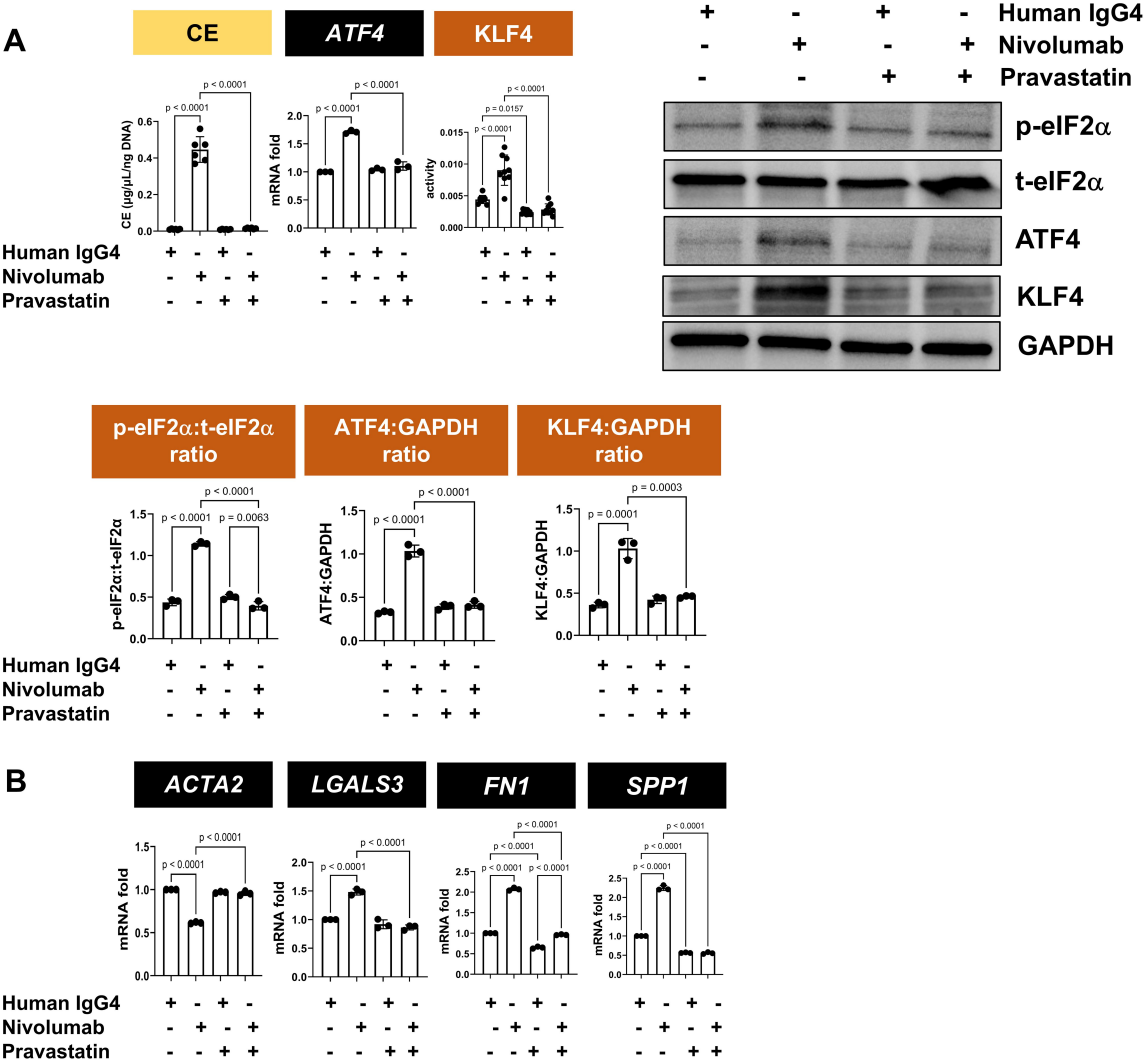
Inhibition of cholesterol biosynthesis blocks nivolumab-induced PERK signaling and SMC phenotypic modulation. **(A,B)** Co-treatment with 200nM pravastatin reverses nivolumab-induced CE accumulation, activation of PERK (eIF2α phosphorylation, *ATF4* expression and protein level, and KLF4 level and activation), downregulation of *ACTA2*, and upregulation of *LGALS3, FN1* and *SPP1* in human SMCs. All data passed normality and were analyzed by 2-way ANOVA followed by Tukey's multiple comparisons test. Nivo - nivolumab, CE – cholesteryl esters.

To confirm SMC phenotypic modulation was dependent on cholesterol biosynthesis, SMCs were exposed to nivolumab or IgG4 isotype in presence of 200nM pravastatin, an HMGCR inhibitor, which effectively blocked CE accumulation, PERK activation (Figure 3A) and SMC phenotypic modulation (Figure 3B).

## Discussion

**Figure 4:**
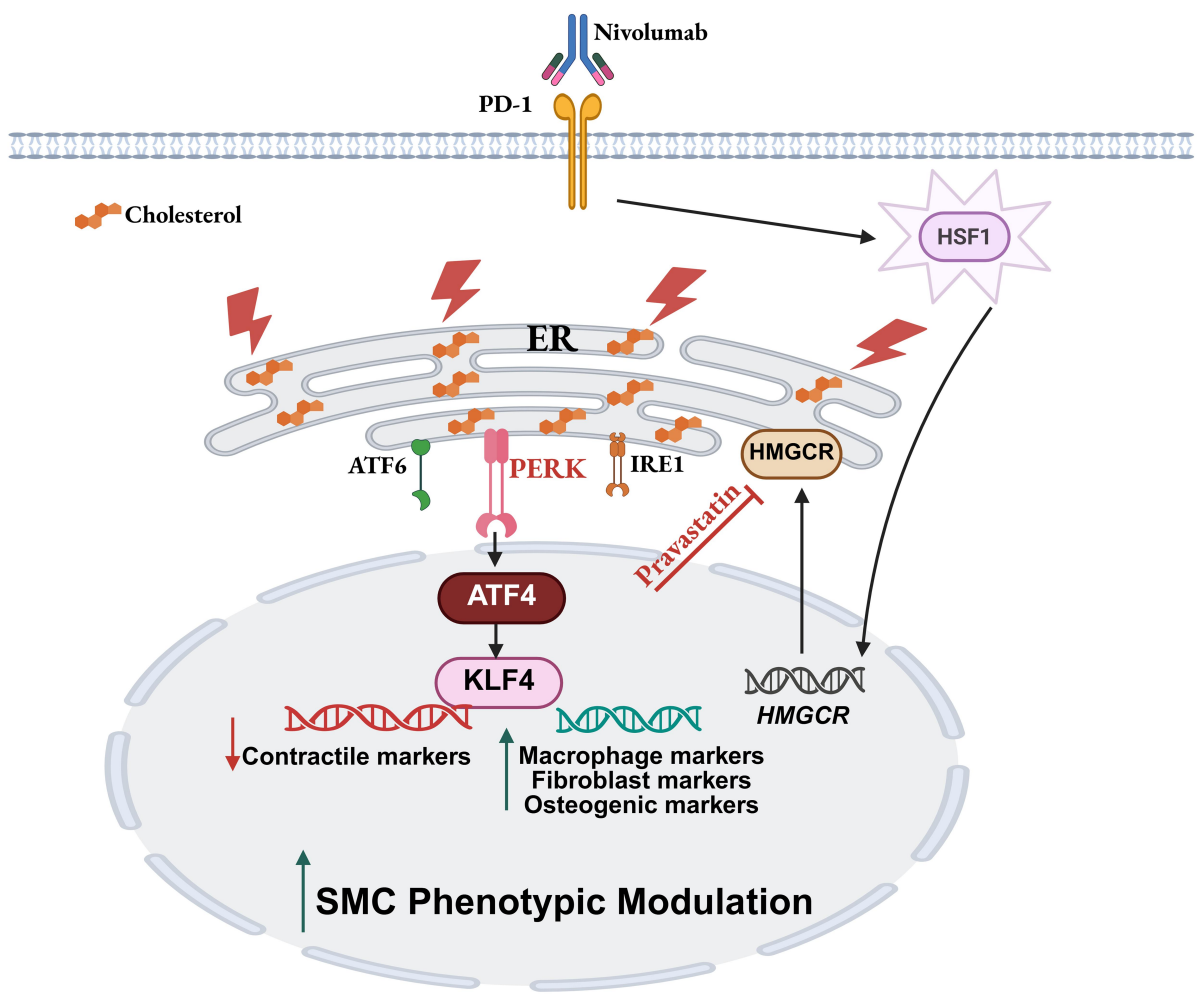
Proposed mechanistic model. Schematic showing that nivolumab exposure activates HSF1, which then drives cholesterol biosynthesis in part through increased HMCGR expression and activation. The increased cholesterol leads to ER stress and PERK activation, and SMC phenotypic modulation, which can be reversed by inhibiting HMGCR with pravastatin treatment.

These data support that PD-1 blockade agents like nivolumab and pembrolizumab, activate SMC cytosolic stress and cholesterol biosynthesis to augment SMC phenotypic modulation, a pathway responsible for ASCVD burden in patients in the absence of hypercholesterolemia (Figure 4) (9). Nivolumab exposure also increases migration and proliferation of SMCs, which can explain, in part, the increased investment of SMCs observed in the atherosclerotic lesions of PD-L1-deficient mice, since migration of medial SMCs into the intima followed by clonal expansion is a well-established mechanism of SMC contribution (5, 16). One limitation of this work is that we were unable to detect PD-1 expression on the surface of SMCs; however, the observation that pre-blocking with an anti-PD-1 antibody but not the corresponding IgG isotype, as well as downregulation of *PDCD1* expression abrogates nivolumab-induced effects on SMCs suggests that the effect involves PD-1 engagement. How nivolumab activates HSF1 remains undefined: nivolumab likely triggers upstream cues of HSF1 activation like reactive oxygen species (ROS) or protein misfolding and aggregation. Additionally, since nivolumab functions by blocking PD-1, it is possible that PD-1 signaling does not directly drive HSF1 signaling; rather, the binding of the antibody itself directly induces cellular stress. Antibody binding can induce crosslinking of surface receptors and disruption of membrane trafficking, which are known to activate stress responses including the cytosolic unfolded protein response and HSF1 (17, 18). It is also possible that the effect of nivolumab on SMCs is not due to canonical PD-1 signaling, but rather a consequence of off-target mechanisms mediated by cell surface receptors like Fc*γ*RIIB, an inhibitory receptor present on B or T cells that binds the Fc region of IgGs and modulates immune cell activation, and whose inhibition in SMCs protects against hypertension and vascular remodeling (19, 20). Rigorous investigation of these molecular pathways is beyond the scope of this manuscript and will be pursued in the future. However, while the precise mechanism by which exposure to an antibody against PD-1 activates HSF1 is unknown, nivolumab-induced SMC modulation can be prevented by blocking cholesterol biosynthesis with statins, which provides a rationale for statin use to prevent ASCVD in ICI-treated patients, even in the absence of hypercholesterolemia. It is notable that ICI-treated patients taking statins due to preexisting hypercholesterolemia have significantly reduced ASCVD disease progression (21). The mechanistic studies presented here are performed in immortalized human SMCs and have yet to be validated *in vivo*; future studies will pursue such validation, and also determine why an ICI antibody induces cytosolic stress in SMCs. At the same time, clinical trials testing the use of statins in ICI-treated patients without hypercholesterolemia should be considered.

## Data Availability

The raw data supporting the conclusions of this article will be made available by the authors, without undue reservation.
